# Role of ion-selective membranes in the carbon balance for CO_2_ electroreduction *via* gas diffusion electrode reactor designs†

**DOI:** 10.1039/d0sc03047c

**Published:** 2020-08-03

**Authors:** Ming Ma, Sangkuk Kim, Ib Chorkendorff, Brian Seger

**Affiliations:** Surface Physics and Catalysis (SurfCat) Section, Department of Physics, Technical University of Denmark 2800 Kgs. Lyngby Denmark brse@fysik.dtu.dk; Surface Chemistry Laboratory of Energy/Electronic Materials (SCHEMA), Department of Chemical Engineering, Pohang University of Science and Technology Pohang 37673 Korea

## Abstract

In this work, the effect of ion-selective membranes on the detailed carbon balance was systematically analyzed for high-rate CO_2_ reduction in GDE-type flow electrolyzers. By using different ion-selective membranes, we show nearly identical catalytic selectivity for CO_2_ reduction, which is primarily due to a similar local reaction environment created at the cathode/electrolyte interface *via* the introduction of a catholyte layer. In addition, based on a systematic exploration of gases released from electrolytes and the dynamic change of electrolyte speciation, we demonstrate the explicit discrepancy in carbon balance paths for the captured CO_2_ at the cathode/catholyte interface *via* reaction with OH^−^ when using different ion-selective membranes: (i) the captured CO_2_ could be transported through an anion exchange membrane in the form of CO_3_^2−^, subsequently releasing CO_2_ along with O_2_ in the anolyte, and (ii) with a cation exchange membrane, the captured CO_2_ would be accumulated in the catholyte in the form of CO_3_^2−^, while (iii) with the use of a bipolar membrane, the captured CO_2_ could be released at the catholyte/membrane interface in the form of gaseous CO_2_. The unique carbon balance path for each type of membrane is linked to ion species transported through the membranes.

## Abstract 2

In this work, the effect of ion-selective membranes on the detailed carbon balance was systematically analyzed for high-rate CO_2_ reduction in GDE-type flow electrolyzers.

## Graphical abstract

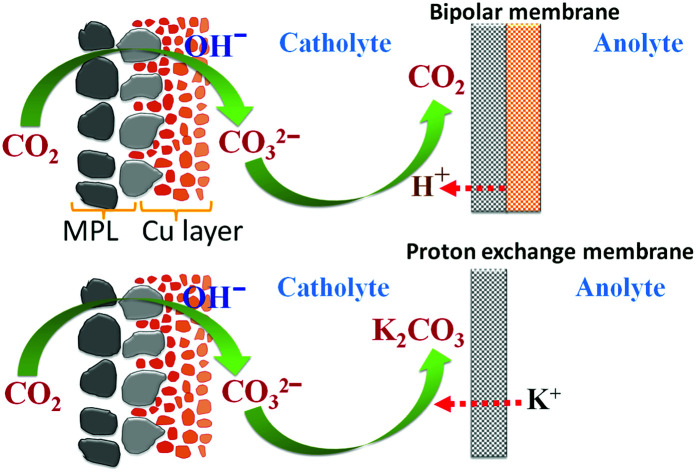

## Introduction

The electrochemical reduction of CO_2_ to valuable chemicals and fuels powered by renewable electricity provides an attractive strategy to close the anthropogenic carbon cycle and store intermittent renewable energy.^1–8^ In the past, great efforts have been devoted to the development of selective, efficient and stable electrocatalysts in CO_2_-saturated aqueous solutions using H-type cells.^9–16^ Striking progress has been made in exploring catalysts for CO_2_ reduction in H-type cells. However, CO_2_ reduction in H-type cells only allows for relatively low current densities due to mass transport limitations in aqueous solutions.^17–19^ Large-scale utilization of electrochemical conversion of CO_2_ requires high reaction rates (*i.e.* high current densities). In this context, flow electrolyzers with gas-diffusion electrodes (GDEs) have gained considerable attention for CO_2_ reduction, owing to the fact that GDEs allow for a very thin mass-transfer boundary layer (∼50 nm).^18,19^ By using GDE-type flow electrolyzers, the mass-transport of CO_2_ and gaseous products on the surface of the catalysts can be accelerated, achieving commercially relevant current densities (>100 mA cm^−2^) along with high selectivity toward a desired product.^20–29^

To date, most of the high-rate CO_2_ reduction studies based on GDE-type flow electrolyzers have been performed using anion exchange membranes (AEMs).^20–29^ However, our recent work demonstrated a substantial crossover of anionic CO_2_ reduction products such as acetate and formate through AEMs in GDE-type flow electrolyzers.^29^ More importantly, after the electrolytes reach a steady state, it was found that about 70% of the consumed CO_2_ is captured at the cathode/electrolyte interface *via* reaction with OH^−^, forming CO_3_^2−^, which is transported to the anolyte *via* an AEM as a charge-carrier.^29^ Subsequently, CO_3_^2−^ coming from the catholyte reacts with H^+^ in the vicinity of the anode, releasing gaseous CO_2_ from the anolyte with the O_2_ stream, which means that most of the consumed CO_2_ (70%) is captured in the catholyte and emitted from the anolyte. In other words, only 30% of the CO_2_ consumed is involved in CO_2_ conversion into products. This finding indicates that many of the current techno-economic analyses for high-rate electroreduction of CO_2_ must be reconsidered if significant CO_2_ crossover occurs.^29,30^

One approach to reduce the CO_2_ crossover would be to use a two-step cascade process, which consists of an initial CO_2_ reduction to CO and a subsequent CO conversion into highly valuable multi-carbon products that have no carbon source crossover.^31,32^ However, even in this two-step procedure with 100% CO faradaic efficiency for the first step, 50% of all consumed CO_2_ could still be emitted out of the anolyte using an AEM.^29^ Theoretically, utilization of a cation exchange membrane (CEM) or a bipolar membrane (BPM) can prevent the CO_2_ crossover in GDE-type flow electrolyzers. However, only a few studies on high-rate CO_2_ reduction (>100 mA cm^−2^) have been carried out in GDE-type electrolyzers using CEMs^33–35^ or BPMs^36–38^ to date.

This study describes a systematic exploration of the effect of ion-selective membranes on the detailed carbon balance including CO_2_ consumption, products and CO_2_ crossover, as well as CO_2_ emission in GDE-type flow electrolyzers. Herein, we demonstrate the comparison of catalytic selectivity, CO_2_ consumption rate (*via* the reaction with OH^−^), and the dynamic change of electrolyte speciation among three different types of ion-selective membranes. By a systematic exploration of the gases released from the catholyte or anolyte, ion species change in the electrolyte and ion species transport *via* membranes, and this work provides mechanistic insights into the role of ion-selective membranes in carbon balancing for high-rate CO_2_ reduction.

## Results and discussion

### Electrocatalytic CO_2_ reduction performance

**Fig. 1:**
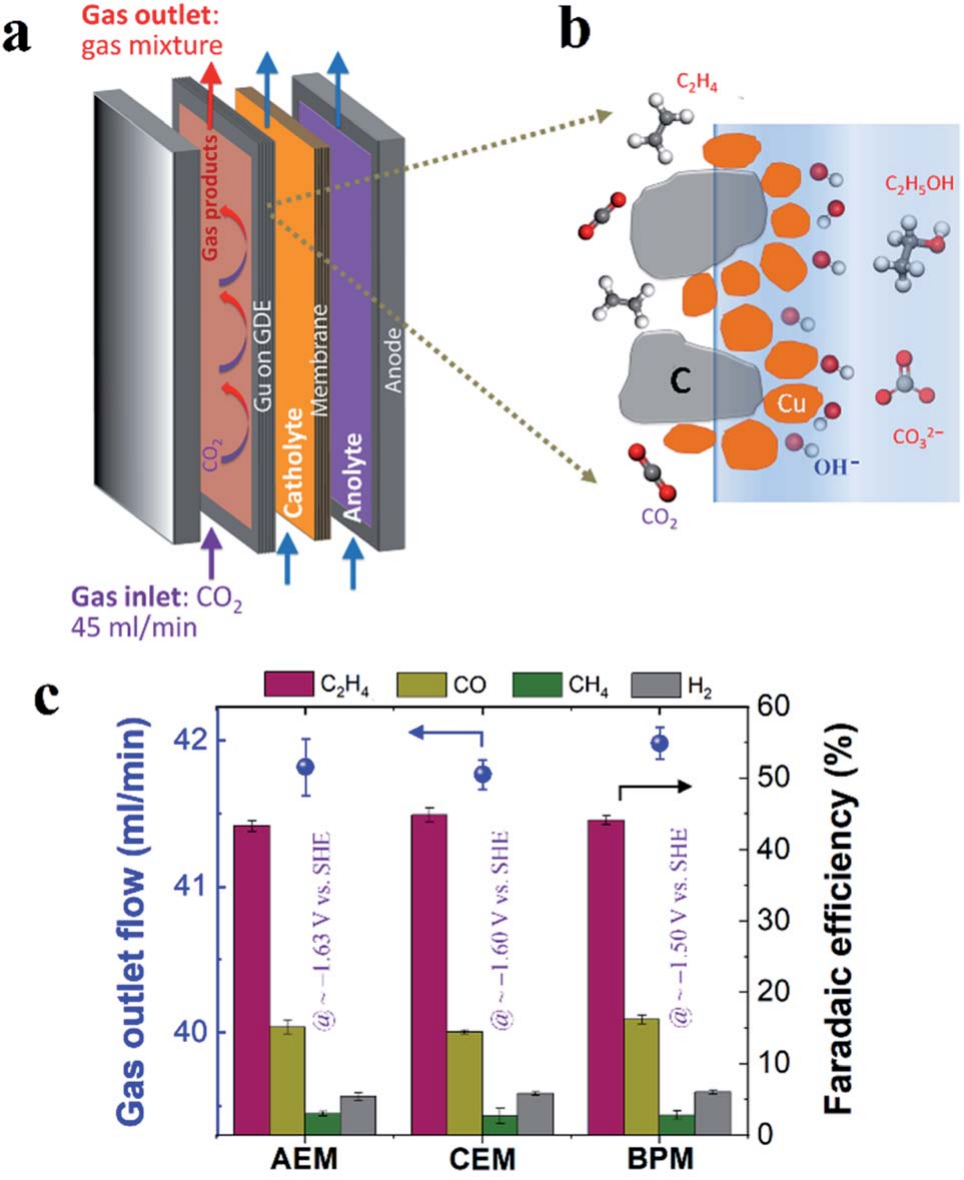
(a) Schematic illustration of three-compartment flow electrolyzers. (b) Schematic illustration of the cathode/electrolyte interface for CO_2_ conversion. (c) Rates of gas flow out of the gas chamber after CO_2_ reduction (left axis) and faradaic efficiencies for gas products (right axis) using different ion-selective membranes in 1 M KHCO_3_ at 200 mA cm^−2^. The *iR*-corrected potentials are labeled with purple color in (c). 45 ml min^−1^ CO_2_ inlet flow was used in all the experiments.

In this work, Cu electrocatalyst layers (∼70 nm) were prepared on top of microporous carbon layers of GDEs by magnetron sputtering at an argon pressure of 2 mTorr (Fig. S1†). The detailed materials characterization of the Cu catalyst layers on GDEs has been reported in our previous work.^29^ We conducted CO_2_ reduction electrolysis experiments in a three-compartment flow electrolyzer where a Cu catalyst coated on a GDE was positioned between the gas and catholyte chambers, as shown in Fig. 1a. An ion-selective membrane was used to separate the catholyte and anolyte flow chambers in which electrolytes continuously flow, and it should be noted that AEM, CEM and BPM were all tested in this work. During CO_2_ reduction, gaseous CO_2_ at a constant flow rate (45 ml min^−1^) was continuously fed into the gas chamber (Fig. 1a), and a fraction of the CO_2_ diffused to the surface of the catalysts in an electrolyte and then converted into gas products such as C_2_H_4_ and liquid products such as ethanol (Fig. 1b). Gas products mixed with the unreacted CO_2_ were directly vented into the gas-sampling loop of a gas chromatograph (GC) for periodic quantification. The liquid products were diluted and circulated in the given catholyte and anolyte reservoirs, and were detected *via* high-performance liquid chromatography (HPLC) after completion of the CO_2_ reduction electrolysis experiments.

In order to get reliable catalytic selectivity for gas products in high-rate CO_2_ reduction, gas flow out of the reactor was monitored *via* a volumetric flowmeter (Fig. S2†).^29^Fig. 1c shows that nearly identical gas flow rates were observed out of the electrolyzer when using an AEM, CEM and BPM in 1 M KHCO_3_ at 200 mA cm^−2^, indicating a similar CO_2_ consumption rate. This observation is primarily due to the same OH^−^ generation rate *via* cathodic reactions (*i.e.* similar local pH created at the cathode/electrolyte interface). The faradaic efficiencies of gas products calculated using these corrected gas flow rates were plotted for different ion-selective membranes (Fig. 1c). As shown in Fig. 1c, C_2_H_4_ is the primary gas product for all the different ion-selective membranes, along with small amounts of CO and H_2_ and only trace amounts of CH_4_. Notably, the faradaic efficiencies for gaseous products had no obvious variation when different types of membranes were utilized (at nearly identical potentials, as shown in Fig. 1c). This result indicates that catalytic selectivity of gaseous products is independent of the type of ion-selective membrane for high-rate CO_2_ reduction in the three-compartment electrolyzers.

**Fig. 2:**
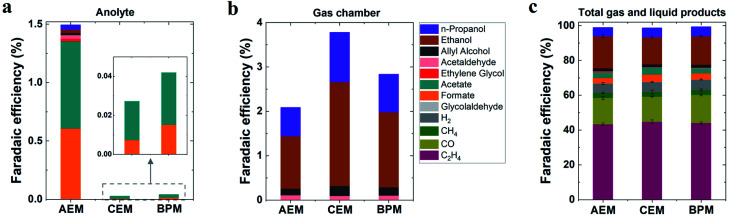
(a) Faradaic efficiencies for detected liquid products in anolyte and (b) faradaic efficiencies for liquid products evaporated from GDEs into the gas chamber. (c) Faradaic efficiencies for all detected gas and liquid products in 1 M KHCO_3_ at 200 mA cm^−2^ for various membranes. Total liquid products were counted *via* analysis of both catholyte and anolyte as well as liquid products evaporated from GDEs into the gas chamber.

In addition to the detected gas products, liquid-phase products in both catholyte and anolyte were all analyzed due to the potential crossover of liquid products from the catholyte to the anolyte *via* membranes.^39,40^ As noted in Fig. 2a, substantial anionic CO_2_ reduction products (such as formate and acetate) crossed over from the catholyte to the anolyte *via* the AEM by electromigration, with only minimal crossover for uncharged liquid products. In contrast, the CEM and BPM exhibited negligible crossover for both anionic liquid products and uncharged products (Fig. 2a). This observation indicates that both CEM and BPM are capable of inhibiting the crossover of anionic and neutral liquid products.

For determining the total amounts of liquid products, liquid products evaporated from GDEs into the gas chamber of the reactor were also collected for analysis (using a setup shown in Fig. S3†).^41^ No matter which type of ion-selective membrane was used, alcohol products such as *n*-propanol and ethanol experienced considerable evaporation through the gas diffusion layer of the GDE (Fig. 2b), which is due to their high volatility. In addition, we found that acetaldehyde had the highest evaporation ratio among liquid products (Fig. S4†). This finding may be attributed to two reasons, (i) its relatively high vapor pressure and (ii) its further reduction to ethanol on the cathode where a substantial amount of acetaldehyde was produced initially and subsequently converted into ethanol.^42^ Based on the quantification of liquid products in both catholyte and anolyte as well as liquid products evaporated from GDEs into the gas chamber (eqn (S12)†), faradaic efficiencies of all liquid products were evaluated for all the different types of membranes (Fig. 2c). As shown in Fig. 2c, ethanol was the dominant liquid product along with *n*-propanol, acetate and formate as minor products. There appears to be no significant variation in liquid product formation across all types of membranes. All the above results imply that the role of ion-selective membrane is almost negligible in affecting catalytic selectivity of high-rate CO_2_ reduction in the three-compartment electrolyzers, owing to the similar local reaction environment created on the cathode *via* the introduction of a catholyte layer. It should be noted that zero-gap electrolyzers lacking a catholyte layer have clearly shown the change of CO_2_ reduction selectivity by the different types of ion-selective membranes.^43,44^

### Capture and emission of CO_2_ throughout the electrolyte

**Scheme 1:**
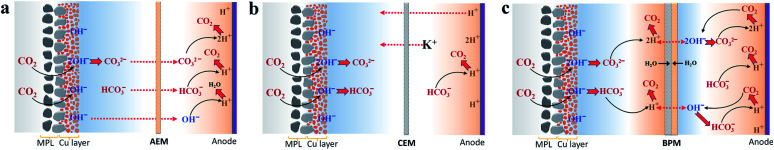
Proposed carbon balance paths *via* CO_2_ capture at the cathode/catholyte interface and CO_2_ evolution from the anolyte or catholyte in flow electrolyzers combined with an AEM (a), CEM (b) and BPM (c), respectively, while using KHCO_3_ as the initial catholyte and anolyte. Red dashed lines with arrows indicate the probable charge-carrying ionic species for membranes. Carbon balance paths for the AEM were adapted from ref. 29.

In accordance with our recent carbon balance study,^29^ the gases released from the anolyte were systematically explored for CO_2_ reduction *via* an AEM with 1 M KHCO_3_, elucidating a two-step procedure of CO_2_ capture at the cathode/electrolyte interface *via* reaction with OH^−^ and subsequent CO_2_ degassing from the anolyte due to H^+^ in the vicinity of the anode (Scheme 1a). With the nearly identical catalytic selectivity (Fig. 2c) and similar total CO_2_ consumption rate (similar gas outlet shown in Fig. 1c), the same OH^−^ generation rate *via* cathodic reactions means that the capability of capturing CO_2_ for carbonate formation at the cathode/electrolyte interface using a CEM and BPM should be similar to that of an AEM. Thus, for a CEM and BPM, substantial additional carbonate anions produced in the reaction of CO_2_ and OH^−^ generated *via* the cathodic reactions must be either balanced with extra cation species (the total anion charge equals the total cation charge) or emitted from the electrolyte as gaseous CO_2_. To uncover the role of different membrane types in the carbon balance for flow electrolyzers, gases released from the electrolyte were detected for the CEM and BPM, respectively (using a closed-cycle anolyte with a vent for gases shown in Fig. S5†).

Theoretically, the composition ratio of CO_2_/O_2_ in the gas stream from the anolyte will be 4, 2 or 0 if the only anion species for neutralizing H^+^ generated on the anode is HCO_3_^−^, CO_3_^2−^ or OH^−^.^28,29^ In addition, under the consideration that HCO_3_^−^, CO_3_^2−^ or OH^−^ is the only anion species of neutralization reaction with H^+^, the theoretically calculated CO_2_ flow rate will be 6.0, 3.0 or 0 ml min^−1^ at 200 mA cm^−2^ with a geometric active area of 2 cm^2^ (Table S2†).

**Fig. 3:**
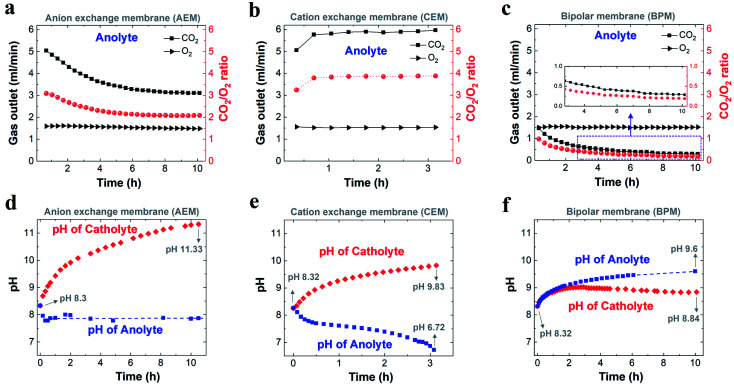
Comparison of flow of CO_2_ and O_2_ released from the anolyte (left axis), and corresponding ratio of CO_2_ to O_2_ (right axis) when using an AEM (a), CEM (b) and BPM (c) over the course of CO_2_ reduction electrolysis at 200 mA cm^−2^. Variation in related electrolyte pH during CO_2_ reduction electrolysis for an AEM (d), CEM (e) and BPM (f), respectively. In all the experiments, 1 M KHCO_3_ was used as the initial catholyte (50 ml) and anolyte (50 ml). (a) and (d) for AEM were adapted based on ref. 29.

Fig. 3a–c show the comparison of gases released in the anolyte over the course of electrolysis for all the different ion-selective membranes. When an AEM was used, the CO_2_/O_2_ ratio decreased from ∼3 to ∼2 in the first 4 h and then remained at ∼2 over the remaining course of electrolysis. This observation is due to the fact that the CO_2_ evolution *via* the H^+^ neutralization reaction changed rapidly from a mixture of HCO_3_^−^ and CO_3_^2−^ to nearly pure CO_3_^2−^ using the AEM (Fig. 3a). In contrast, as noted in Fig. 3b, the CEM experienced a consistent CO_2_/O_2_ ratio of ∼4 and a constant CO_2_ flow rate of 6 ml min^−1^ for the duration of electrolysis at 200 mA cm^−2^, which implies that the CO_2_ formation was always derived from HCO_3_^−^ in the anolyte. This finding is ascribed to the fact that the CO_3_^2−^ formed *via* CO_2_ capture in the catholyte cannot be transported to the anolyte *via* the CEM since the functional groups (typically SO_3_^−^ groups) only allow cation species (such as K^+^) to pass through (Scheme 1b). It should be noted that the CO_2_ reduction electrolysis *via* the CEM was tested for just ∼3 h, since the anolyte conductivity rapidly decreased from ∼70 mS cm^−1^ to ∼3 mS cm^−1^ after ∼3 h (Fig. S8b†), which is consistent with previous work.^45^ All the above results with the CEM indicate that almost no anionic species were transported to the anolyte *via* the membrane, but cation species such as K^+^ served as the main charge carrier *via* the CEM. Thus, the concentration of KHCO_3_ in the anolyte was significantly reduced over time as K^+^ was constantly transported to the catholyte and the remaining HCO_3_^−^ in the anolyte was consumed for CO_2_ evolution (Scheme 1b).

A bipolar membrane is composed of a cation exchange layer (CEL) and an anion exchange layer (AEL) as well as a catalyst layer that is sandwiched between the CEL and AEL. The catalyst layer in a BPM dissociates water (fed from both the catholyte and anolyte) into H^+^ and OH^−^, which is subsequently transported to the catholyte and anolyte *via* the CEL and AEL, respectively (Scheme 1c).^46^ With the use of a BPM (Fig. 3c), the flow rate of CO_2_ released from the anolyte rapidly decreased from 1.4 ml min^−1^ to 0.5 ml min^−1^ in the first 4 h, corresponding to a decline in the CO_2_/O_2_ ratio from ∼1 to ∼0.3. This observation may be linked to the fact that an alkaline boundary layer near the AEL of the BPM created *via* the constant supply of OH^−^ from the BPM was unfavorable for releasing CO_2_ (the distance between the anode and the membrane was ∼3 mm). In addition, the almost constant conductivity in both catholyte and anolyte over the 10 h electrolysis (Fig. S8c†) may imply that neither anionic species (CO_3_^2−^ or HCO_3_^−^) nor cationic species (K^+^) had any apparent crossover. This result reveals that the additional anion species (CO_3_^2−^ or HCO_3_^−^) generated by CO_2_ capture could not be accumulated in the catholyte during CO_2_ reduction electrolysis due to the charge balance issue (the total anion charge must equal the total cation charge). Thus, the additional CO_3_^2−^ or HCO_3_^−^ should be emitted from the catholyte as gaseous CO_2_. As expected, gas bubbles released from the catholyte were observed when a BPM was used (no gas evolution was observed in the catholyte using an AEM or CEM), and this gas evolution immediately disappeared after stopping the electrolysis.

**Fig. 4:**
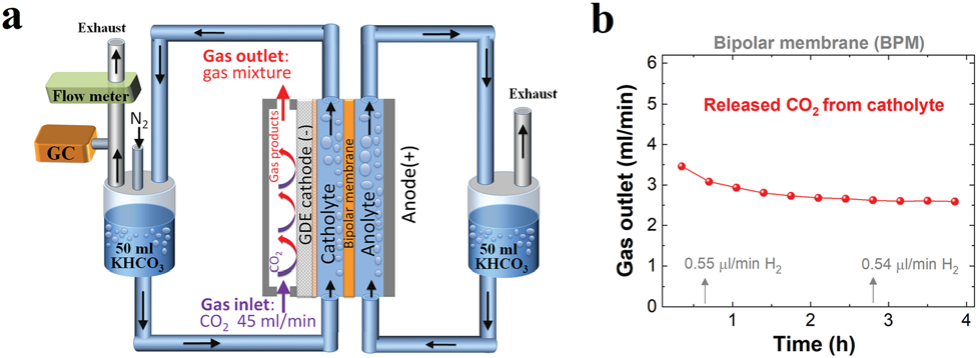
(a) Schematic illustration of the flow cell setup for detecting gases released from the catholyte over the course of CO_2_ reduction when using a BPM (N_2_ with a constant flow rate was used as a carrier gas). (b) Flow rate of CO_2_ released from the catholyte when using a BPM for CO_2_ reduction at 200 mA cm^−2^ with a negligible amount of H_2_. 1 M KHCO_3_ was used as the initial catholyte (50 ml) and anolyte (50 ml).

To verify the CO_2_ degassing in the catholyte, the gases released from the catholyte during the CO_2_ reduction electrolysis were analyzed using a setup shown in Fig. 4a. Fig. 4b shows CO_2_ degassing from the catholyte when using a BPM, owing to the neutralization reaction of CO_3_^2−^ or HCO_3_^−^ with H^+^ near the CEL of the BPM (Scheme 1c), which is in line with previous BPM work.^37^ In addition, the related flow rate of CO_2_ released from the catholyte slightly decreased from ∼3.5 ml min^−1^ to ∼2.6 ml min^−1^, and was maintained at ∼2.6 ml min^−1^ over the electrolysis experiment (Fig. 4b). This observation can be attributed to the fact that the carbon source (anion species) for CO_2_ evolution abruptly transformed from a mixture of HCO_3_^−^ and CO_3_^2−^ to almost pure CO_3_^2−^. In addition, a fraction of CO_2_ released from the catholyte chamber can transport to the cathode surface to be reused for both CO_2_ reduction^37^ and the buffering reaction with OH^−^ at the cathode/electrolyte interface. This back-diffusion effect leads to a slightly lower CO_2_ flow (∼2.6 ml min^−1^) compared to the theoretical value (3.0 ml min^−1^). Furthermore, with nearly identical catalytic selectivity (Fig. 2c) and the same OH^−^ generation rate on the cathode (due to the same current density) among all the different membranes, the utilization of a fraction of CO_2_ released from a catholyte with a BPM results in a slightly lower CO_2_ consumption rate in the gas chamber. This result is in line with the slightly higher gas outlet flow rate for the BPM in comparison with those of the AEM and CEM (Fig. 1c).

While each type of ion-selective membrane had a different flow rate of CO_2_ released from the anolyte, O_2_ was detected with a constant flow rate of ∼1.5 ml min^−1^ during the electrolysis irrespective of membrane type (Fig. 3a–c). This finding is consistent with the theoretical value of the O_2_ flow rate (1.5 ml min^−1^ shown in Table S2†) at 200 mA cm^−2^ for a geometric active area of 2 cm^2^.

To further understand the transformation of anionic species in the electrolyte, the pH of the electrolyte was also monitored over the course of the electrolysis for all the membranes. Fig. 3e shows that for a CEM the catholyte pH was enhanced from 8.3 to nearly 9.8 after ∼3 h. The catholyte pH with the AEM increased to 10.2 after ∼3 h under identical conditions. Thus, the similar increasing trend in catholyte pH between the AEM and CEM over 3 h indicates that the captured CO_2_ at the cathode/electrolyte interface (*via* reaction with OH^−^) mainly formed CO_3_^2−^ using the CEM,^29^ leading to CO_3_^2−^ acting as the dominant anion species in the catholyte after 3 h. The catholyte pH with the BPM was maintained below 9 over the entire electrolysis experiment (Fig. 3f) due to the fact that a constant supply rate of H^+^ from water dissociation in the BPM enables carbonate and bicarbonate concentrations in the catholyte to reach a steady sate. In addition, this pH < 9 also indicates that most of the existing anion species in the catholyte was bicarbonate over the entire electrolysis (Table S4†). However, the observed CO_2_ flow rate (2.6 ml min^−1^) from the catholyte (after reaching a steady state) also reveals that CO_2_ was captured and converted to CO_3_^2−^ at the cathode/electrolyte interface, and then combined with the aforementioned H^+^ at the BPM/catholyte interface to release CO_2_. In addition, it should be noted that the theoretical calculations have shown that the pH near the cathode is ∼13 in 1 M KHCO_3_ at 200 mA cm^−2^,^18^ which means that the reaction of CO_2_ with OH^−^ at the cathode/electrolyte interface forms CO_3_^2−^ instead of HCO_3_^−^ (eqn (S8) and (S9)†). Thus, all these results reveal that the CO_2_ captured by the electrolyte near the cathode formed CO_3_^2−^ irrespective of membrane type.

We found that the anolyte quickly reached a near neutral pH for both the AEM and the CEM during the electrolysis (Fig. 3d and e), which allows for CO_2_ degassing in the anolyte. Specifically, the anolyte pH with the AEM was maintained at ∼7.9 after 20 min (Fig. 3d), owing to the fact that the constant H^+^ generation rate near the anode and continuous carbonate supply derived from the catholyte created a steady state for all the anion species in the anolyte *via* the neutralization reactions (Scheme 1a). In contrast, with the CEM, the anolyte pH rapidly decreased from 8.3 to 6.7 over 3 h (Fig. 3e). This finding is due to the fact that the CO_2_ degassing with the continuous consumption of KHCO_3_ in the anolyte created a CO_2_-saturated KHCO_3_ anolyte and its concentration gradually reduced over time (pH of CO_2_-saturated 0.1 M KHCO_3_ is ∼6.8). Interestingly, a slow increase in the anolyte pH from 8.3 to 9.6 was observed over 10 h electrolysis when using the BPM, as shown in Fig. 3f. This observation may be linked to a slow variation in the anionic species concentrations (here, an increase in the CO_3_^2−^/HCO_3_^−^ ratio was likely created) in the anolyte during the electrolysis. This slow alteration is ascribed to the fact that the anolyte species did not completely reach a steady-state within 10 h electrolysis *via* the two major reactions, (i) the reaction of CO_2_ with OH^−^ at the BPM/anolyte interface forming CO_3_^2−^/HCO_3_^−^, and (ii) simultaneously, CO_3_^2−^/HCO_3_^−^ converting into CO_2_ near the anode (Scheme 1c).

### Carbon balance *via* different types of membranes and implications

**Fig. 5:**
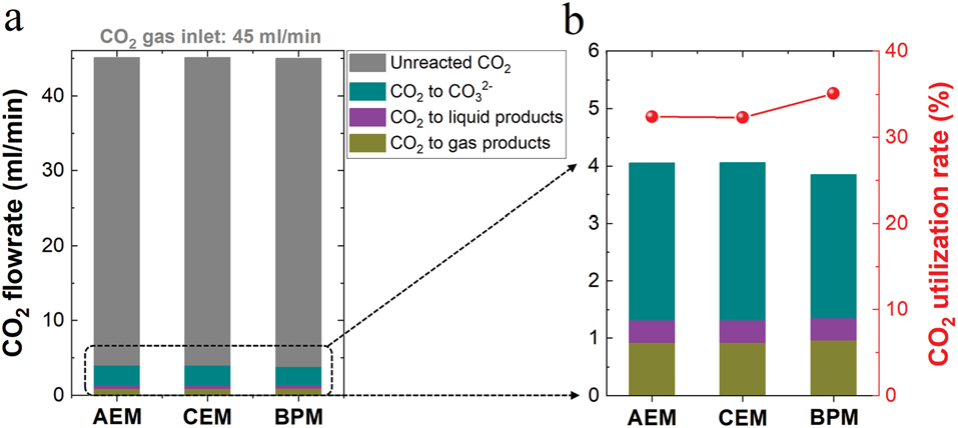
(a) Carbon balance for high-rate CO_2_ reduction in 1 M KHCO_3_ using different membranes. The unreacted (*i.e.* residual) CO_2_ flow rate as well as the total consumed CO_2_ flow rate for carbonate formation and CO_2_ reduction to all liquid and gas products were considered. (b) Ratio of CO_2_ converted into products to total CO_2_ consumption (right axis).

For high-rate CO_2_ reduction in flow electrolyzers, the carbon source for CO_2_ fed from the inlet of the reactor must be balanced with that of all CO_2_ reduction products, CO_2_ captured by electrolyte (carbonate formation) and residual CO_2_ out of the reactor (*i.e.* unreacted CO_2_). As noted in Fig. 5a, (i) the flow rate of residual unreacted CO_2_ out of the reactor, (ii) the flow rate of CO_2_ consumed for carbonate formation *via* the reaction with OH^−^ (*i.e.* captured CO_2_ throughout the electrolyte) and (iii) the flow rate of consumed CO_2_ that was converted into all the gaseous and liquid products add up to a total CO_2_ flow rate of ∼45 ml min^−1^ for each type of ion-selective membrane. Thus, the carbon element during the electrolysis is balanced with that of the CO_2_ inlet flow rate (45 ml min^−1^) in this work. In addition, Fig. 5b shows the nearly identical CO_2_ consumption rate for the formation of gaseous and liquid products using different ion-selective membranes, which is in line with the roughly same catalytic selectivity shown in Fig. 2c.

It should be noted that there should be nearly the same carbonate formation rate (*via* CO_2_ reaction with OH^−^) near the cathode among all the different membranes due to the identical OH^−^ generation rate *via* cathodic reactions at identical current densities. While membrane types should have a minimal effect on the total carbonate formation rate near the cathode, the BPM had a slightly lower consumption rate of CO_2_ from the gas chamber for carbonate formation compared to those of the AEM and CEM, as shown in Fig. 5b. This finding correlates with the discrepancy in carbon balance paths among the three different types of membranes. In other words, while the unavoidable CO_2_ capture near the cathode forms carbonate in the catholyte, the end result of where the carbonate goes is different in each type of membrane. For the CEM, the captured CO_2_ was accumulated in the form of carbonate in the catholyte without emission. In contrast, when the AEM was used, the captured CO_2_ in the form of carbonate crossed over to the anolyte and was emitted as gaseous CO_2_ with the O_2_ stream in the anolyte. Notably, with the BPM, the captured CO_2_ could be released from the catholyte as gaseous CO_2_. Thus, a fraction of the generated CO_2_ in the catholyte may be involved in the reaction with OH^−^ for carbonate formation, which corresponds to a relatively low consumption rate of CO_2_ in the gas chamber for carbonate formation (∼65% of the total CO_2_ consumption), as shown in Fig. 5b. In addition, the reuse of a fraction of the released CO_2_ in the catholyte, derived from the captured CO_2_ in the form of carbonate, also results in a slightly higher CO_2_ utilization rate of the BPM (ratio of CO_2_ converted into products *versus* total CO_2_ consumption) in Fig. 5b.

From an economic and environmental perspective, the released CO_2_ from the electrolyte in flow electrolyzers would need to be captured and recycled. When the AEM is used, the released CO_2_ in the anolyte can only be recycled for CO_2_ reduction after removing O_2_ in the gas mixture (mole ratio of CO_2_/O_2_ is 2 : 1). Interestingly, the BPM could degas CO_2_ from the catholyte, which can be directly fed into the gas compartment for CO_2_ conversion due to its high purity (∼100% CO_2_ by mole). Thus, compared to the necessary CO_2_ and O_2_ separation process for CO_2_ recycling with the AEM, the BPM has the potential to reduce the total cost of the carbon source. However, it should be noted that using a BPM for high-rate CO_2_ reduction (current densities > 100 mA cm^−2^) currently requires an additional potential (>∼1.5 V) for membranes that may reduce the energy efficiency of CO_2_ conversion reactors.^47^ In this work, an additional potential of ∼2 V was observed when using the BPM at 200 mA cm^−2^ (Fig. S9†). Thereby, how to balance the energy efficiency along with the easy recyclability of the produced CO_2_ in the catholyte (from inevitably captured CO_2_) with the use of BPMs will need a full techno-economic analysis in the future.

## Conclusions

In conclusion, our results show that the role of ion-selective membranes is minimal in affecting the catalytic selectivity of high-rate CO_2_ reduction, owing to the nearly same local reaction environment created near the catalysts through having a catholyte layer. By rigorously analyzing gases released from electrolytes as well as monitoring electrolyte pH, we found that most of the consumed CO_2_ source (≥∼65%) was captured *via* reaction with OH^−^ near the cathode to form CO_3_^2−^, which is almost independent of membrane type.

Importantly, each type of ion-selective membrane produces a unique carbon balance path for the captured CO_2_ source. Specifically, the captured CO_2_ in the form of CO_3_^2−^ could cross an AEM from the catholyte to the anolyte and then be emitted as gaseous CO_2_ mixed with the O_2_ stream. In contrast, the captured CO_2_ could not be transported to the anolyte when using a CEM or BPM. With a CEM, captured CO_2_ in the form of carbonate continuously accumulated in the catholyte, since there was no concomitant H^+^ supply for CO_2_ evolution (mainly K^+^ crossed the membrane). With the bipolar membrane, the captured CO_2_ was released from the catholyte as gaseous CO_2_, owing to the reaction of carbonate with H^+^ transported from its cation exchange layer. In addition, while for an AEM CO_2_ was emitted together with O_2_, for a BPM the pure CO_2_ was released, which can be directly recycled back to the gas compartment for CO_2_ conversion, correspondingly decreasing the cost of the CO_2_ source. This study shows that while the catalytic selectivity is independent of the type of ion-selective membrane, membrane type plays an important role in the corresponding carbon balance path for high-rate CO_2_ reduction. Thus, future work should focus on membrane exploration for achieving the practical utilization of high-rate CO_2_ reduction.

## Author contributions

M. M. and B. S. developed the conceptual idea, designed the experiments and wrote the original manuscript. S. K. carried out a part of the electrolyte pH and conductivity measurements. All authors contributed to discussing the results and editing the manuscript.

## Conflicts of interest

There are no conflicts to declare.

## Supplementary Material

SC-011-D0SC03047C-s001
