# Supplementary material for: Role of ion-selective membranes in the carbon balance for CO2 electroreduction via gas diffusion electrode reactor designs
Source: Chem Sci. 2020 Aug 3;11(33):8854–61. doi: 10.1039/d0sc03047c (PMC8163407; doi:10.1039/d0sc03047c)
Supplement: SC-011-D0SC03047C-s001 [file SC-011-D0SC03047C-s001.pdf]

Electronic Supplementary Material (ESI) for Chemical Science.
This journal is © The Royal Society of Chemistry 2020

SUPPLEMENTAL INFORMATION

Role of Ion-Selective Membranes in the Carbon Balance for CO_2 Electroreduction via Gas Diffusion Electrode Reactor Designs

Ming Ma,[†] Sangkuk Kim,[†,‡] Ib Chorkendorff[†] and Brian Seger[†,*]

[†]Surface Physics and Catalysis (Surfcat) Section, Department of Physics, Technical University of Denmark, 2800 Kgs Lyngby, Denmark

[‡]Surface Chemistry Laboratory of Energy/Electronic Materials (SCHEMA), Department of Chemical Engineering, Pohang University of Science and Technology, Pohang 37673, Korea

*Author to whom correspondence should be addressed.

E-mail address: brse@fysik.dtu.dk

Tel.: +45 45253174

Materials

Potassium bicarbonate (≥99.95%) purchased from Sigma Aldrich was used in this study without further purification. Anion exchange membrane (AEM, Fumasep FAA-3-PK-75), cation exchange membrane (Nafion™ 212), bipolar membrane (BPM, Fumasep FBM) and gas-diffusion electrode (GDE, Sigracet 39 BC) were purchased from Fuel Cell Store. Iridium dioxide (IrO_2) purchased from Dioxide Materials was used as an anode in flow electrolyzers of high-rate CO_2 reduction.

Catalysts preparation

For obtaining high purity Cu catalsyts, Cu layers were prepared on top of microporous layer of gas-diffusion electrodes by direct current magnetron sputtering (50 W) from a Cu target at an argon pressure of 2 mTorr. Figure 1S shows a typial schematic illustration for Cu depostion using magnetron sputtering under an argon ambient. The energetic Ar^+ ions are created in a glow discharge plasma, thus Ar^+ bombardment occurs on the cathode Cu target, which leads to the removal of Cu atoms. Subseqently, the sputtered Cu atoms condense on a substrate (i.e. GDE) to form a Cu layer. In this work, with ~4 nm/min Cu deposition rate, the thickness of the Cu layers were controlled by the deposition time.

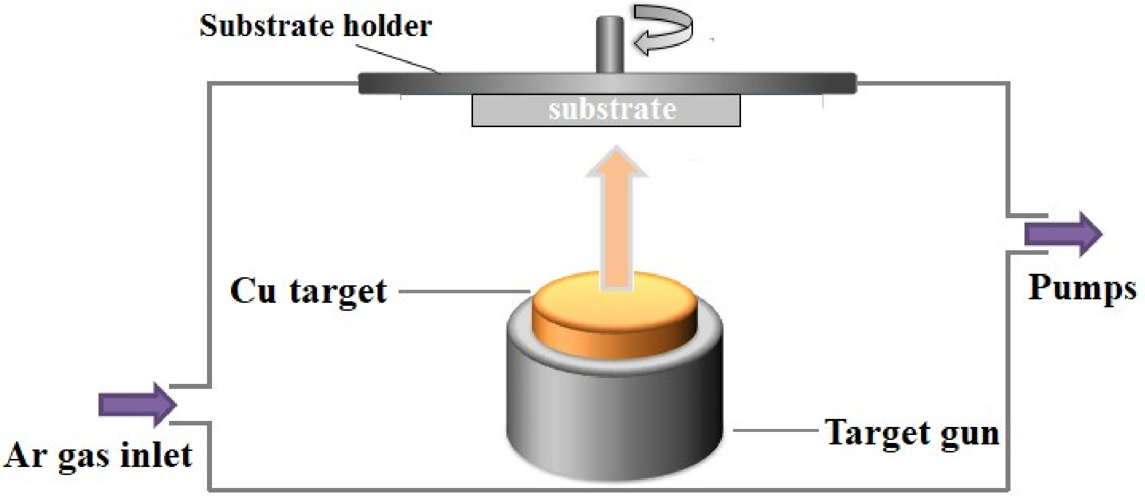

Figure S1. Schematic illustration of magnetron sputtering for Cu deposition from a Cu target.

High local pH near the cathode

During the process of electrochemical reduction of CO_2, CO_2 and H_2O can be electrochemically converted into a variety of gaseous products such as CO, C_2H_4 and CH_4 on the surface of the catalyst in electrolytes based on the below reactions[1–4]:

$$CO_2 + H_2O + 2e^- \rightarrow CO + 2OH^- \quad \text{(-0.11 V vs. RHE)} \quad \text{(S1)}$$

$$CO_2 + 6H_2O + 8e^- \rightarrow CH_4 + 8OH^- \quad \text{(0.17 V vs. RHE)} \quad \text{(S2)}$$

$$2CO_2 + 8H_2O + 12e^- \rightarrow C_2H_4 + 12OH^- \quad \text{(0.08 V vs. RHE)} \quad \text{(S3)}$$

In addition to gaseous products, liquid such as ethanol, formate and acetate also can be produced on the surface of the catalyst in aqueous solutions, as follows:

$$CO_2 + H_2O + 2e^- \rightarrow HCOO^- + OH^- \quad \text{(-0.03 V vs. RHE)} \quad \text{(S4)}$$

$$2CO_2 + 5H_2O + 8e^- \rightarrow CH_3COO^- + 7OH^- \quad \text{(-0.26 V vs. RHE)} \quad \text{(S5)}$$

$$2CO_2 + 9H_2O + 12e^- \rightarrow C_2H_5OH + 12OH^- \quad \text{(0.09 V vs. RHE)} \quad \text{(S6)}$$

In the electrocatalytic CO_2 reduction process, the competing H_2 evolution is an unavoidable reaction. The electroreduction of H_2O to H_2 on the surface of catalyst according to the reaction[1]:

$$2H_2O + 2e^- \rightarrow H_2 + 2OH^- \quad \text{(0 V vs. RHE)} \quad \text{(S7)}$$

Due to the above cathodic reactions (Equation (3-9), a large amount of OH^- ions can be created at the cathode/electrolyte interface at high current densities, which creates a significantly higher pH near the surface of cathode compared to that of the bulk solutions.[2,3]

CO_2 capture via high local pH

During CO_2 reduction in flow electrolyzers using 1 M $KHCO_3$, CO_2 from the gas chamber of the electrolyzers reacts with OH^- ions produced at the cathode/electrolyte interface accoding to the below reactions of CO_2 and OH^-:

$$OH^- + CO_2 \leftrightarrow HCO_3^- \quad \text{(pKa=7.8*)} \quad \text{(S8)}$$

$$OH^- + HCO_3^- \leftrightarrow CO_3^{2-} + H_2O \qquad \text{(pKa=10.3)} \qquad \text{(S9)}$$

* This is at a CO_2 partial pressure of 1 bar in 1 M HCO_3^-.[5]

CO_2 reduction performance

The electrocatalytic reduction of CO_2 was peformed in a three-chamber flow electrolyzer made from Teflon at ambient temperature and pressure. At the reactor, an ion-selective membrane was used to sperate catholyte and anolyte flow compartments. Catholyte and anolyte bottles were filled with 50 ml 1 M $KHCO_3$, respectively (Figure S2). In addition, a fixed geometric surface area (2 cm^2) of Cu layer was used for all the tests in this study.

CO_2 was purged into gas compartment at a constant flowrate of 45 ml/min, and then a fraction of gaseous CO_2 diffuses to the surface of the catalyst in electrolyte for CO_2 conversion. In addtion, CO_2 also can be captured to form carbonate (equation S8 and S9) via the reaction of CO_2 with OH^- generated at the cathode/electrolye inteface.[5] Thus, the high CO_2 conversion rate to gas (C_2) and liquid products as well as high local pH can lead to a substantial CO_2 consumption at high current densities, correspondingly varying the gas flow (gas mixture) out of the reactor. The volumetric flowrate of gas outlet (gas mixture) after reactor was monitored by flow meter in CO_2 reduction (Figure S2), and then faradaic efficiencies of gas products were evaluated under the consideration of gas flow variation between inlet and outlet. It should be noted that the average catalytic selevitity of gas products during 2.5 h CO_2 reduction electrolysis was used in this work.

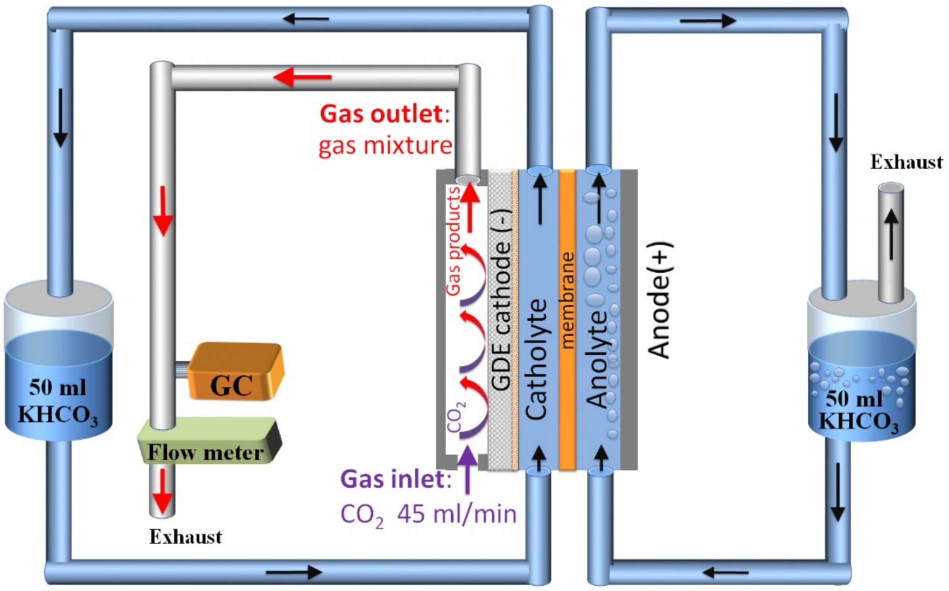

Figure S2. Schematic illustration of flow cell setup for electrocatalytic CO_2 reduction.

Gas and liquid product analysis

Gas products mixing with unreacted CO_2 flowed out of the electrolyzers, directly injecting into the gas-sampling loop of a gas chromatography (PerkinElmer, Clarus® 590). Ar was used as a carrier gas with a contant flowrate of 10 sccm. The gas chromatography was equipped with a packed Molecular sieve 13x column and a packed Hayesep Q column to separate the gas products. Thus, H_2, CO, CH_4 and C_2H_4 could be identified at different reaction times using a thermal conductivity detector. In addition, the peak area of each gas product was compared to standards (calibration gases) to determine the corresponding concentration of gaseous products. Thus, we can get the faradaic efficiency of a certain gas product as follows:

$$FE\ (\%) = \frac{n \times C_{gas\ product} \times \emptyset t \times \frac{P_o}{RT} \times F}{I \times t} \times 100\%$$

$$= \frac{n \times C_{gas\ product} \times \emptyset \times \frac{P_o}{RT} \times F}{I} \times 100\% \qquad (S10)$$

where n is the number of electrons required for producing one molecule of the related gas product, and $C_{gas\ product}$ is the concentration of gas product measured by GC (here, $C_{gas\ product}$ is

the mole fraction of gas product in the total gas outlet mixture). $\emptyset$ and t is the gas flowrate out of the electrolyzers and the electrolysis time, respectively. P_o is the ambient pressure, R is the ideal gas constant, T is the absolute temperature, F is Faraday constant, and I is the applied current.

The liquid-phase products are analyzed after the electrolysis using a high performance liquid chromatography (HPLC, Agilent 1200 series). Liquid-phase products were separated by an Aminex HPX-87H column (Bio-Rad) that was maintained at 50 °C for the duration of the detection. The HPLC was equipped with a diode array detector (DAD) and a refractive index detector (RID), and the signal response of the DAD and RID was calibrated by known concentration solutions. Thus, we can get the concentration of the detected liquid-phase product. The faradaic efficiency of liquid products can be calculated by equation:

$$FE\ (\%) = \frac{n \times C_{liquid\ product} \times V \times F}{I \times t} \times 100\% \qquad (S11)$$

where n is the number of electrons required for producing one molecule of the related liquid product, and $C_{product}$ is the molar concentration of gas product measured by HPLC. V is the volume of the electrolyte. To obtain accurate selectivity of liquid products, we measured the volume of catholyte and anolyte after electrolysis, respectively.

Collection of liquid from electrolyte

It should be noted that the ion species carried with water molecules (hydrated ion) transports via membrane, which means the volume of catholyte and anolyte was vaired after electrolysis. For AEM, a decrease in catholyte volume was observed with increased anolyte volume after several hours of CO_2 reduction electrolysis, because of the transportion of the anion species hydrated with water molecules from catholyte to anolyte via AEM as charge carriers. In contract, the use of CEM experienced an inceased catholyte volume with correspondingly decreased anolyte over the course of electrolysis, due to that the cation species hydrated water molecules transported from anolyte to catholyte via AEM as charge carriers. Notably, no obvious variation in both cathlyte and anolyte when BPM was used, which is due to that water supplied almost equally from both catholye and anolyte was disociated into H^+ and OH^-, transporting to catholyte and anolyte, respectively.

Based on the aforementioned discussion, in order to obtain accurate selectivity of liquid products, volume of catholyte and anolyte was also measured for each test after electrolysis, respectively.

Collection of liquid products evaporated from GDEs

Some liquid products can be evaporated from the gas diffusion layer of GDE and then flow out of the gas compartment of the reactor with unreacted CO_2 and gas products. To collect the evaporated liquid products from GDEs (i.e. gas chamber), gas outlet flow after the reactor was directly purged into a sealed bottle filled with 30 ml de-ionized water (the outlet flow tube was immersed into de-ionized water), as shown in Figure S3. After completion of CO_2 reduction, the liquid products diluted with de-ionized water in that sealed bottle were analysed via high-performance liquid chromatography (HPLC). Figure 2b presents the faradaic efficiencies of liquid products evaporated from GDEs when using distinct ion-selective membranes, indicating that only alcohols products such as ethanol and propanol evaporate and escape from the cathode/electrolyte interface irrespective of membrane types, which is due to their high volatility.

In addition, both catholyte and anolyte in the given reservoirs were collected for quantification of liquid products, owing to liquid products crossover from catholyte to anolyte via membranes.[5] Thus, the total amount of one certain liquid product formed on cathode GEDs can be written as:

$$N_{total\ liquid} = N_{liquid\ in\ anolyte} + N_{liquid\ in\ catholyte} + N_{evaporated\ liquid} \quad \text{(S12)}$$

where $N_{liquid\ in\ anolyte}$ and $N_{liquid\ in\ catholyte}$ are the amount of one certain liquid product detected in anolyte and catholyte, respectively. $N_{evaporated\ liquid}$ is the amount of one certain liquid product evaporated from GDEs. Here, the evaporation ratio of one certain liquid product formed on cathode GDEs can be calculated based on the below equation:

$$Evaporation\ ratio\ (\%) = \frac{N_{evaporated\ liquid}}{N_{total\ liquid}} \times 100\% \quad (S13)$$

Thus, the equation (S13) was used to calculate a ratio between the amount of one certain liquid product evaporated from GDEs and the total amount of corresponding liquid product formed on the cathode, as shown in Figure S4.

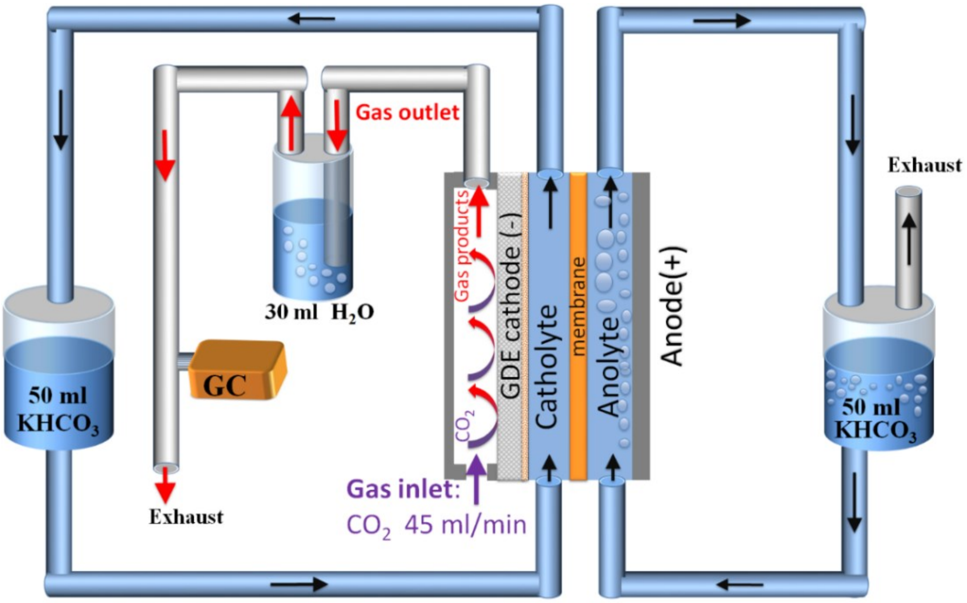

Figure S3. The schematic illustration of flow cell setup for collecting liquid products evaporated from GDEs during CO_2 reduction.

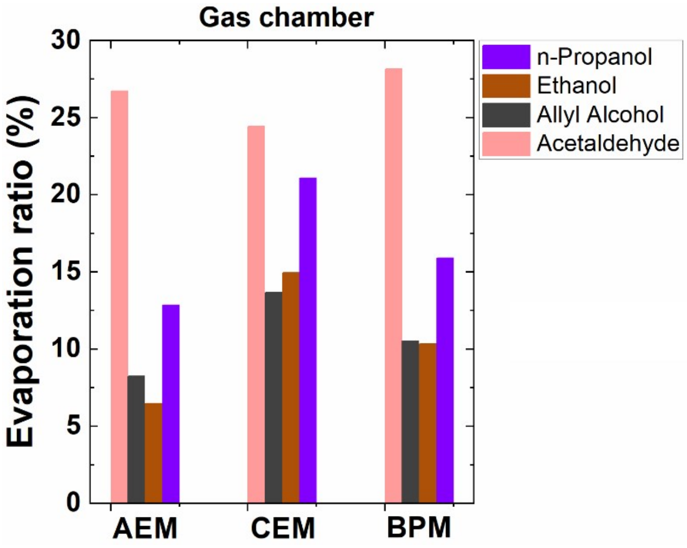

Figure S4. Evaporation ratio of related liquid products escaped from GDEs (i.e. gas chamber) at 200 mA/cm^2 when using AEM, CEM and BPM, respectively.

Analysis of gas released from the anolyte

When the electrocatalytic CO_2 reduction occurs on the surface of the cathode, water oxidation reaction (i.e. O_2 evolution) takes place on the anode surface. By the water oxidation reaction, a large amount of H^+ can be created at the anode/electrolyte interface, which leads to a decrease of pH locally near the anode. Subsequently, H^+ produced at the anode/electrolyte interface can be neutralized with HCO_3^-, CO_3^{2-} or OH^- in anolyte. The H^+ neutralization with HCO_3^- or CO_3^{2-} forms CO_2, leading to CO_2 degassing from anolyte with the stream of O_2.[5] For analysing the gases released from anolyte over the course of CO_2 reduction, the flow electrolyser setup in Figure S5 was utilized. In that setup, N_2 at a constant flowrate was used as a carrier, thus gases released from anolyte were diluted with N_2, directly venting into the gas sampling-loop of the GC for periodical quantification. The volumetric gas flow released from anolyte was also monitored by in situ flow meter over the electrolysis, as shown in Figure S5.

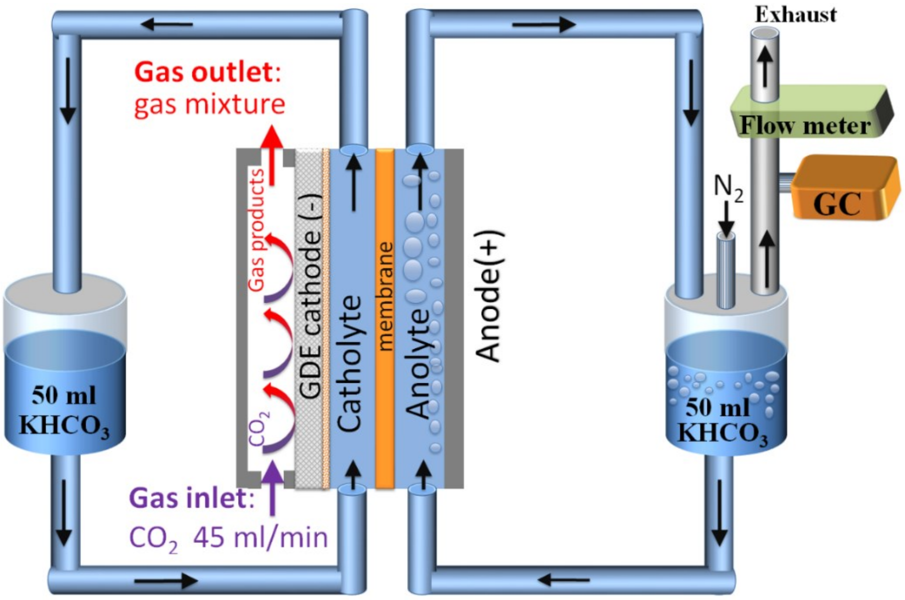

Figure S5. The schematic illustration of flow cell setup for detection of gases released from the anolyte over the course of CO_2 reduction.

Analysis of gas released from the catholyte using BPMs

No any gas evolution in catholtye was observed when AEM or CEM was used. However, under the use of bipolar membrane in flow electrolyzers, we found that gas bubbles released from the catholyte, which is unique in comparison with the other two membranes. To analyze the gas released from catholyte over the course of CO_2 reduction using BPM, a test setup in Figure S6 was utilized. Similar to gas analysis from anolyte, a constant N_2 flow was also used as a carrier gas, which mixed with gases released from catholyte, venting into the gas sampling-loop of the GC for periodical quantification, followed with an in situ volumetric flow meter (Figure S6). We found CO_2 gas releasd from the cathlyte, along with only trace amount of H_2 in Figure 4. It should be noted that the mole ratio of CO_2/H_2 released from the catholyte is 5000, which means that the purity of released CO_2 is about 99.98%.

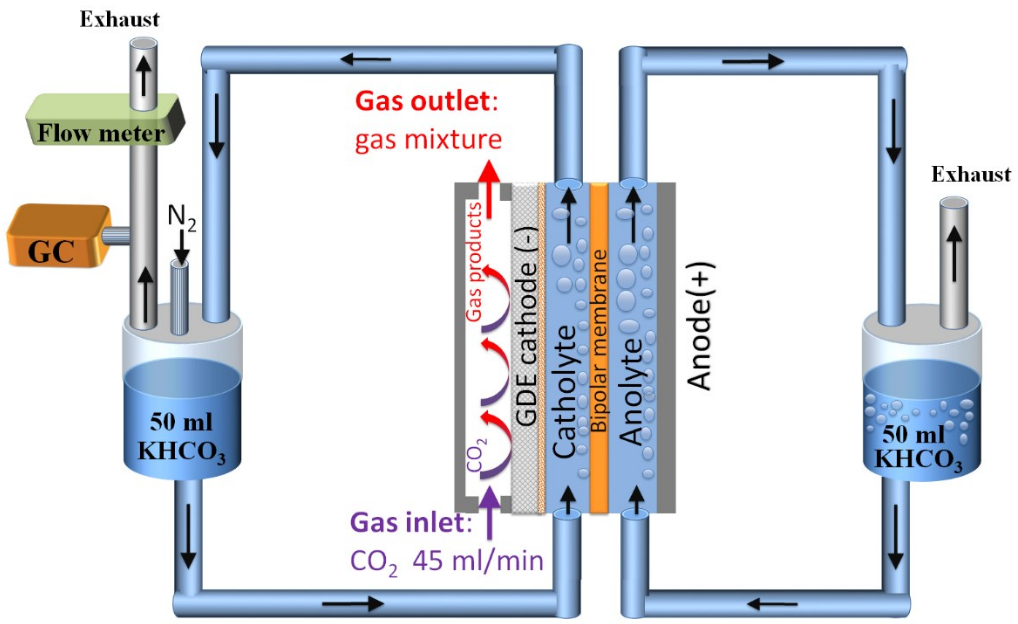

Figure S6. The schematic illustration of flow cell setup for detection of gases released from the catholyte over the course of CO_2 reduction under the use of BPM. 1 M $KHCO_3$ was used as initial catholyte (50 ml) and anolyte (50 ml).

Applied potentials on the cathode

Potentiostatic electrochemical impedance spectroscopy (PEIS) was conducted on Cu deposited GDE in the flow electrolyzer to determine the solution resistance (Rs). A detailed procedure was described in a previous work.[5] It should be noted that the distance between reference and cathode was less than 2 mm in order to reduce Rs in this work. Table S1 shows the solution resistance for the different ion-selective membranes. However, the cathodic reactions at high current densities can lead to a significant change of ion species and related concentration in the vicinity of the cathode, which indicates a difference in solution resistance near the cathode at high current densities compared to that of PEIS which was performed at relative low current densities. Thus, this difference in the solution resistance is closely correlated with the accuracy in IR-corrected potentials at a high current.

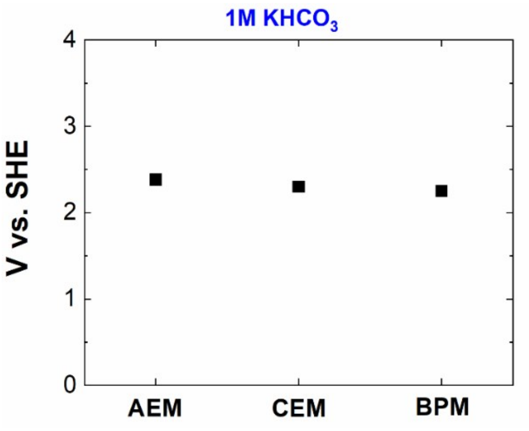

Figure S7. Applied potentials under the use of the different ion-selective membranes in 1 M $KHCO_3$ aqueous solutions. (The potentials were not iR-corrected).

Table S1. IR-corrected potentials for CO_2 reduction at 200 mA/cm^2 in 1 M $KHCO_3$ under the use of the different ion-selective membranes.

Membrane type	Rs (Ω)	Corrected V vs. SHE
AEM	1.375	-1.63
CEM	1.25	-1.60
BPM	1.38	-1.50

Theoretical estimation of O_2 and CO_2 flowrate generated from electrolyte

Assuming that all charge passed through the anode is just employed for oxidation of water into O_2, thus theoretical O_2 flowrate released from anolyte can be expressed as[5]:

$$\emptyset\,(O_2) = \frac{Q_{tot}}{nF} \times \frac{RT}{P_o} \qquad (S14)$$

where n and Q_{tot} are the number (here is 4) of electrons lost from 2 H_2O for forming one O_2 molecule and totoal charge passed through the anode, respectively. F is the faradaic constant, R is ideal gas constant, T is absolute temperature, and P_o is ambient pressure.

From our prevous work, the ratio of CO_2 and O_2 released from the anolyte will be 4, 2 and 0 if the only anion species for neutralization reaction with H^+ is HCO_3^-, CO_3^{2-} or OH^-.[5] Thus, after

getting the O_2 flowrate at 200 mA/cm^2 (the cathode with 2 cm^2 geometric active area was used for all the tests) based on the equation S14, we can easily get the related flow of CO_2, as shown in Table S2.

Table S2. The theoretically calculated flowrates of O_2 and CO_2 released from electrolyte if the only anion species for neutralization reaction with H^+ is HCO_3^-, CO_3^{2-} or OH^- at 200 mA/cm^2.

Anion species for neutralization with H^+	CO_2 flow (ml/min)	O_2 flow (ml/min)
HCO_3^-	5.970	1.492
CO_3^{2-}	2.985	1.492
OH^-	0	1.492

Electrolyte pH and conductivity measurements

pH of the catholyte and the anolyte was monitored by a pH meter (pH 110, VWR) during the electrolysis. In addition, the pH meter was also equipped with a temperature sensor for the temperature-compensation. The pH meter was calibrated by a standard pH 7 buffer and a standard pH 10 buffer before the measurement.

The conductivity of the catholyte and the anolyte was monitored by a conductivity meter (PCE-PHD 1-PH, PCE Instruments) during CO_2 reduction electrolysis. Before the measurement, the conductivity meter was calibrated via conductivity standard of 1413 μS / cm (25 °C; 0.01 M KCl) and 111.8 mS / cm (25 °C; 1 M KCl) purchased from VWR. It should be noted that both of the calibration and the measurement were temperature-compensated due to that the solution conductivity is also temperature-dependent at a fixed solution concentration.

Because the AEMs are positively charged, anions can pass, and the crossover of positively charged ions will be decreased drastically.[4] In other words, no ideal AEM can absolutely avoid the crossover of positively charge ions. In this work, for the use of the AEM, when the anion species served as the main charge carriers transporting from the catholyte to the anolyte via the membrane, a small amount of K^+ slowly crossed over from the anolyte to the catholyte via the AEM. Thus, the anolyte concentration gradually decreased, along with the correspondingly increased catholyte concentration over electrolysis, leading to the related variation in the anolyte and catholyte conductivity over time (Figure S8a).

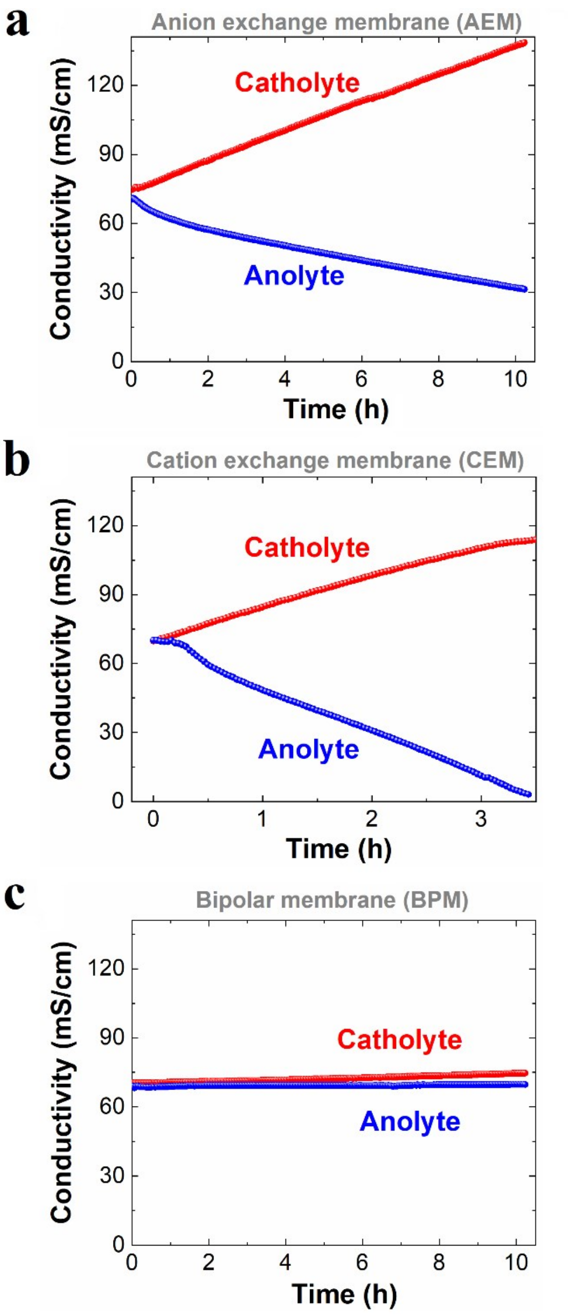

Figure S8. Conductivity of catholyte and anolyte as a function of time when using AEM (a), CEM (b) and BPM (c) over the course of CO_2 reduction electrolysis at 200 mA/cm^2. 1 M $KHCO_3$ was used for both catholyte (50 ml) and anolyte (50 ml).

Calculation of the carbon balance

The residual unreacted CO_2 flowrate in the gas outlet (gas mixture) out of gas compartment of flow electrolzyers can be written as:

$$\emptyset_{residual\,CO_2} = \emptyset_{outlet} - (\emptyset_{CO} + \emptyset_{CH_4} + \emptyset_{C_2H_4} + \emptyset_{H_2}) \qquad (S15)$$

where $\emptyset_{outlet}$ is the monitored gas flowrate out of the ractor during CO_2 reduction electrolysis using the setup shown in Figure 2S. Here, $\emptyset_{CO}$, $\emptyset_{CH_4}$, $\emptyset_{C_2H_4}$ and $\emptyset_{H_2}$ are the gas flowrate of CO, CH_4, C_2H_4 and H_2 produced from electrochecmial CO_2 conversion in the gas outlet, respectively. Based on the equation S1-3, each molecule of CO, CH_4 and C_2H_4 formation requires 1, 1 and 2 CO_2 molecule, Thus, the consumed CO_2 flowrate that is converted into all gas products (CO, C_2H_4 and CH_4) in CO_2 reduction can be expresed as below:

$$\emptyset_{CO_2\,to\,gas} = \emptyset_{CO} + \emptyset_{CH_4} + 2\emptyset_{C_2H_4} \qquad (S16)$$

Depending on the number of carbon atoms in liquid molecule produced in CO_2 reduction, the consumed CO_2 flowrate involved in all liquid products formation can be written as:

$$\emptyset_{CO_2\,to\,liquid} = \emptyset_{C_1} + \emptyset_{C_2} + \emptyset_{C_3} \qquad (S17)$$

where $\emptyset_{C_1}$, $\emptyset_{C_2}$, and $\emptyset_{C_3}$ are the consumed CO_2 flowrate for forming C_1, C_2 and C_3 liquid products, respectively. For high-rate CO_2 reduction, the inevitably caputred CO_2 in forms of carbonate via reaction with OH^- could consume substantial CO_2 flow, significantly reducing the total gas flow out of the reator (Figure 1c). It is known that the carbon element from CO_2 inlet flowrate should be eventually balanced with those of residual unreacted CO_2, all products and carbonate formed via reaction between OH^- and CO_2. Thus, the consumed CO_2 flowrate via the reaction with OH^- generated on the cathode surface can be expressed as:

$$\emptyset_{OH^-} = \emptyset_{inlet\,CO_2} - (\emptyset_{residual\,CO_2} + \emptyset_{CO_2\,to\,gas} + \emptyset_{CO_2\,to\,liquid}) \qquad (S18)$$

where $\emptyset_{inlet\,CO_2}$ is CO_2 flowrate fed into the gas chamber of the reactor. In this work, a constant CO_2 flow was used. It should be noted that mass flow controller used in this work was calibrated for CO_2 flow by volumetric flow meter before and after each CO_2 reduction test for high accuracy. Thus, we got the below carbon balance in Table S3.

Table S3. Carbon balance and related CO_2 utilization rate (ratio of CO_2 used in products formation versus total CO_2 consumption) for different ion-selective membranes in 1 M $KHCO_3$.

Membrane type	$\emptyset_{CO_2\ to\ gas}$ (ml/min)	$\emptyset_{CO_2\ to\ liquid}$ (ml/min)	$\emptyset_{OH^-}$ (ml/min)	$\emptyset_{residual\ CO_2}$ (ml/min)	CO_2 utilization rate (%)
AEM	0.922	0.3917	2.74	41.02	~32.4
CEM	0.91885	0.391	2.75	40.99	~32.3
BPM	0.966	0.385	2.499	41.15	~35.1

($\emptyset_{CO_2\ to\ gas}$: the consumed CO_2 flowrate that is converted into all gas products (CO, C_2H_4 and CH_4) in CO_2 reduction; $\emptyset_{CO_2\ to\ liquid}$: the consumed CO_2 flowrate for all liquid products in CO_2 reduction (such as ethanol); $\emptyset_{OH^-}$: the consumed CO_2 flowrate via the reaction with OH^- generated in cathodic reactions; $\emptyset_{residual\ CO_2}$: the residual unreacted CO_2 flowrate in the gas outlet (gas mixture) out of gas compartment of flow electrolzyers)

Correlation between the carbonate/bicarbonate ratio and pH

The pH of a buffer solution can be estimated by the Henderson–Hasselbalch equation, as below:

$$pH = pK_a + log_{10}(\frac{[CO^{2-}_3]}{[HCO^-_3]}) \quad (S19)$$

where pKa is the acid dissociation constant, and $[CO_3^{2-}]/[HCO_3^-]$ is the concentration ratio of CO_3^{2-} to HCO_3^-. The above equation can be rewritten as:

$$\frac{[CO^{2-}_3]}{[HCO^-_3]} = 10^{(pH - pK_a)} \quad (S20)$$

Here, the pKa is 10.3 at 25 °C based on the equation S9. The pH of the catholyte was measured by the pH meter for the different membranes, as shown in Figure 3d, e and f. Thus, the concentration ratio of carbonate/bicarbonate in the catholyte can be estimated according to the equation S20. Table S4 shows the typical concentration ratio of carbonate to bicarbonate at different pH values. Before starting the electrolysis, the initial 1 M $KHCO_3$ has a pH value of ~8.3, which corresponds to $[CO_3^{2-}]/[HCO_3^-]$ ratio of 0.01. For the AEM, the pH is ~11.33 after ~10 h (Figure 3d), corresponding to $[CO_3^{2-}]/[HCO_3^-]$ ratio of 10, which is consistent with the catholyte transformation from bicarbonate to carbonate over the electrolysis. For the BPM, the catholyte pH was maintained < 9 over the entire electrolysis (Figure 3f), indicating that most of anion species was bicarbonate.

Table S4. The calculated ratio of $[CO_3^{2-}]/[HCO_3^-]$ at typical catholyte pH values.

pH	$[CO_3^{2-}]/[HCO_3^-]$ ratio
~8.3	0.01
~9	0.05
~10	1
~11.33	10

Cell voltage

Cell voltage as a function of time when using the three different membranes over the course of CO_2 reduction electrolysis is shown in Figure S9, which indicates that an additional potential of ~ 2 V was required for the BPM compared to those of the use of CEM and AEM. In addition, it should be noted that an apparent fluctuation in the applied potentials was observed due to the effect of CO_2 degassing at the BPM/catholyte interface. With the use of the CEM, the anolyte conductivity rapidly decreased from ~70 mS/cm to ~3 mS/cm after ~ 3 h (Figure S8b), which leads to the increased cell voltages for maintaining the constant current density of 200 mA/cm^2 (Figure S9b).

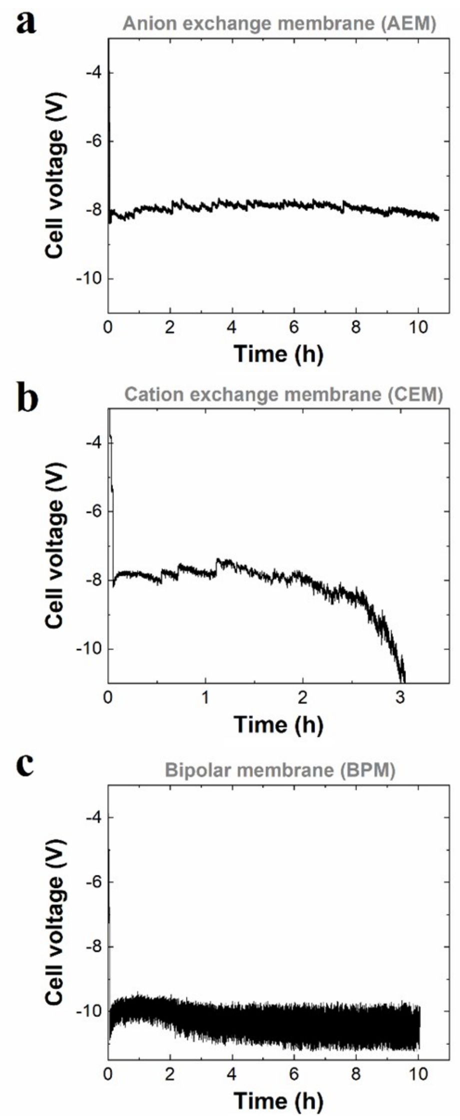

Figure S9. Cell voltage as a function of time when using AEM (a), CEM (b) and BPM (c) over the course of CO_2 reduction electrolysis at 200 mA/cm^2. 1 M KHCO3 was used for both catholyte (50 ml) and anolyte (50 ml). The distance between the Cu cathode and the membrane was ~15 mm.

REFERENCES

1 K. P. Kuhl, T. Hatsukade, E. R. Cave, D. N. Abram, J. Kibsgaard and T. F. Jaramillo, *Journal of the American Chemical Society*, 2014, **136**, 14107–14113.

2 Y. Hori, in *Modern Aspects of Electrochemistry*, ed. E. Vayenas, C. G., White, R. E., Gamboa-Aldeco, M. E., Springer New York, New York, NY, 2004, vol. 70, pp. 89–189.

3 M. Ma, K. Djanashvili and W. A. Smith, *Angewandte Chemie International Edition*, 2016, **55**, 6680–6684.

4 K. P. Kuhl, E. R. Cave, D. N. Abram and T. F. Jaramillo, *Energy & Environmental Science*, 2012, **5**, 7050.

5 M. Ma, E. L. Clark, K. T. Therkildsen, S. Dalsgaard, I. Chorkendorff and B. Seger, *Energy & Environmental Science*, 2020, **3**, 977–985.
